# New Aspects of the Interplay between Penicillin Binding Proteins, *murM*, and the Two-Component System CiaRH of Penicillin-Resistant Streptococcus pneumoniae Serotype 19A Isolates from Hungary

**DOI:** 10.1128/AAC.00414-17

**Published:** 2017-06-27

**Authors:** Inga Schweizer, Sebastian Blättner, Patrick Maurer, Katharina Peters, Daniela Vollmer, Waldemar Vollmer, Regine Hakenbeck, Dalia Denapaite

**Affiliations:** a Department of Microbiology, University of Kaiserslautern, Kaiserslautern, Germany; b Centre for Bacterial Cell Biology, Institute for Cell and Molecular Biosciences, Newcastle University, Newcastle upon Tyne, United Kingdom

**Keywords:** Streptococcus pneumoniae, PBP2x, PBP1a, CiaH, MurM, peptidoglycan analysis

## Abstract

The Streptococcus pneumoniae clone Hungary^19A^-6 expresses unusually high levels of β-lactam resistance, which is in part due to mutations in the MurM gene, encoding a transferase involved in the synthesis of branched peptidoglycan. Moreover, it contains the allele *ciaH232*, encoding the histidine kinase CiaH (M. Müller, P. Marx, R. Hakenbeck, and R. Brückner, Microbiology 157:3104–3112, 2011, https://doi.org/10.1099/mic.0.053157-0). High-level penicillin resistance primarily requires the presence of low-affinity (mosaic) penicillin binding protein (PBP) genes, as, for example, in strain Hu17, a closely related member of the Hungary^19A^-6 lineage. Interestingly, strain Hu15 is β-lactam sensitive due to the absence of mosaic PBPs. This unique situation prompted us to investigate the development of cefotaxime resistance in transformation experiments with genes known to play a role in this phenotype, *pbp2x*, *pbp1a*, *murM*, and *ciaH*, and penicillin-sensitive recipient strains R6 and Hu15. Characterization of phenotypes, peptidoglycan composition, and CiaR-mediated gene expression revealed several novel aspects of penicillin resistance. The *murM* gene of strain Hu17 (*murM*_Hu17_), which is highly similar to *murM* of Streptococcus mitis, induced morphological changes which were partly reversed by *ciaH232. murM*_Hu17_ conferred cefotaxime resistance only in the presence of the *pbp2x o*f strain Hu17 (*pbp2x*_Hu17_). The *ciaH232* allele contributed to a remarkable increase in cefotaxime resistance in combination with *pbp2x*_Hu17_ and *pbp1a* of strain Hu17 (*pbp1a*_Hu17_), accompanied by higher levels of expression of CiaR-regulated genes, documenting that *ciaH232* responds to PBP1a_Hu17_-mediated changes in cell wall synthesis. Most importantly, the proportion of branched peptides relative to the proportion of linear muropeptides increased in cells containing mosaic PBPs, suggesting an altered enzymatic activity of these proteins.

## INTRODUCTION

Streptococcus pneumoniae is a commensal bacterium that colonizes the human nasopharynx (1, 2). Moreover, it is a leading respiratory human pathogen, causing a variety of diseases mainly in children, elderly people, and immunocompromised patients (3, 4). S. pneumoniae has long been considered a highly β-lactam-susceptible organism. However, high-level penicillin-resistant S. pneumoniae (PRSP) strains frequently express multiple antibiotic resistance genes, and their incidence has increased dramatically since the 1980s worldwide. Only a few clones of serotypes 14, 23F, 19F, 19A, 9V, and 6B that spread globally were mainly responsible for this scenario (5). The introduction of a 7-valent pneumococcal conjugated vaccine in 2000 followed by a 13-valent vaccine in 2010 was associated with a decrease in the incidence of infections due to PRSP but was accompanied by the appearance of antibiotic-resistant clones expressing nonvaccine serotypes (6–8). This development underlines the importance of continuing surveillance for antibiotic resistance and furthering our understanding of resistance mechanisms.

Resistance to β-lactams in S. pneumoniae is primarily driven by alterations in the transpeptidase domain of three penicillin binding proteins (PBPs), PBP2x, PBP2b, and PBP1a (for a review, see reference 9). These altered PBPs display a decrease in affinity to the β-lactam antibiotics, while the alterations apparently leave the enzyme function unaffected. The DNA sequences of PBP genes in penicillin-sensitive S. pneumoniae strains are well conserved. In contrast, the PBP genes of PRSP strains contain mosaic sequence blocks of different lengths that may differ by over 20% at the DNA level or 10% at the amino acid level compared with the sequences of the corresponding regions in the PBP genes of penicillin-sensitive S. pneumoniae strains (see reference 9 and references within). There is evidence that these mosaic blocks were acquired from commensal mitis group streptococci, especially S. mitis, via homologous recombination (10–13). Subsequent intraspecies recombination and mutations lead to the further diversification of PBP genes, resulting in a large number of PBP alleles (14, 15).

PBP2x and PBP2b are essential proteins and represent the primary target of β-lactams (for a review, see reference 16). Mutations in each of these proteins result in low-level β-lactam resistance and can be selected in sensitive strains (17–19). In contrast, mutations in PBP1a do not result in a detectable resistance increase if they are not accompanied by PBP2x and/or PBP2b alterations (20). PBP2b does not interact with third-generation cephalosporins, such as cefotaxime; therefore, it is not a target for this class of compounds (21). Thus, PBP2b mediates resistance primarily to penicillins, such as piperacillin, whereas high-level cefotaxime resistance requires only alterations in PBP2x in combination with alterations in PBP1a (for reviews, see references 9 and 22).

PBPs are crucial enzymes acting in the biosynthesis of peptidoglycan (PG), a major cell wall component that surrounds the cytoplasmic membrane and is required to maintain the shape and osmotic stability of bacteria. Pneumococcal PG forms a multilayered network of glycan chains of alternating *N*-acetylglucosamine (Glc*N*Ac) and *N*-acetylmuramic acid (Mur*N*Ac) residues connected via short stem peptides consisting of l-Ala–γ-d-iGln–l-Lys–d-Ala–d-Ala (23). Unamidated glutamate is prevalent only in monomers, indicating that the transpeptidases require fully amidated peptide substrates (24). The cross-linking of stem peptides, the crucial reaction that leads to the network structure of PG, is catalyzed by the d,d-transpeptidase activity of PBPs. In S. pneumoniae, the stem peptides can be further modified by replacement of the l-Lys ε-amino group with a dipeptide consisting of l-Ala or l-Ser followed by an invariable l-Ala residue, resulting in branched peptides (24–26). Overall, the pneumococcal PG consists of a complex mixture of mainly monomeric, dimeric, and trimeric peptides with modifications in the glycan and peptide chains (24). Some PRSP clones contain increased levels of branched muropeptides due to the presence of a mosaic MurM gene (25, 27, 28) whose product, MurM, displays increased catalytic activity (29). Hence, it was proposed that low-affinity PBPs from PRSP isolates prefer branched peptides as the substrate (27), but experimental evidence is still lacking. Remarkably, the deletion of *murM* (also called *fibA*) in PRSP results in a complete loss of the resistance phenotype (30–32), a phenomenon that is not understood in molecular terms.

In addition to the PBP and MurM genes, other genes have been implicated in the β-lactam resistance of S. pneumoniae (for a review, see reference 9). The first non-PBP gene identified in spontaneous β-lactam-resistant S. pneumoniae laboratory mutants was *ciaH* (33, 34), encoding the histidine kinase CiaH, part of the two-component regulatory system CiaRH (33, 35). Mutations in CiaH result in higher levels of expression of genes regulated by the cognate response regulator CiaR and confer a pleiotropic phenotype: an increase in the level of β-lactam resistance, prevention of competence development, and protection from lysis-inducing conditions (35–37). A functional CiaRH system is required for proper growth in laboratory mutants containing altered PBP2x (20, 37). Distinct mutations in *ciaH* were also identified in clinical isolates of S. pneumoniae, including isolate Hungary^19A^-6 (36, 38). The *ciaH* alleles from laboratory mutants of the S. pneumoniae R6 strain strongly enhance expression of the CiaR regulon, whereas CiaH variants of clinical strains increase the activity of CiaR-dependent promoters only moderately under the same circumstances (36).

The CiaRH system, part of a complex regulatory network, is ubiquitously present among nonpyogenic streptococci (39, 40). The response regulator CiaR directly controls 15 promoters that drive the transcription of 29 genes; among these are 5 genes specifying small noncoding *cia*-dependent small RNAs (csRNAs) (35, 41). The csRNAs feed into another regulatory network, including the competence regulon (42). Therefore, it is not surprising that the CiaRH system affects a variety of physiological processes, such as β-lactam resistance (33, 36), genetic competence (33, 37, 43–45), bacteriocin production (46, 47), the maintenance of cell integrity (37, 48), and host colonization (49). The CiaRH system was shown to be highly active and nearly constitutive under a variety of laboratory conditions (50) and in animal models of colonization and virulence (49, 51–53), but the signal detected by the sensor kinase CiaH is still unknown.

High-level penicillin- and multiple-antibiotic-resistant serotype 19A S. pneumoniae strains were prevalent in Hungary during the 1990s (54, 55) and occurred in the Czech Republic and Slovakia as well (56). These type 19A isolates express a PBP3 with an electrophoretic mobility different from that of most other S. pneumoniae isolates and differ in their PBP2x sequences, accompanied by a variable PBP profile (57, 58), and their genomes appear to be surprisingly variable (59). Accordingly, multilocus electrophoretic typing revealed several electrophoretic types (57). Multilocus sequence typing (MLST) identified one major clone, Hungary^19A^-6 (representative strain HUN663, also named Hungary^19A^-6), of sequence type (ST) 268 (ST268) (5). In this work, we studied the resistance determinants in two serotype 19A strains from Hungary, strains Hu15 and Hu17. They represent members of the same clone, ST226, a single-locus variant of Hungary^19A^-6 differing in the *ddl* allele, encoding the d-Ala–d-Ala ligase (60). Strain Hu15 is penicillin sensitive, whereas strain Hu17 exhibits high-level penicillin resistance; their MIC values for benzylpenicillin are 0.06 μg/ml and 24 μg/ml, respectively (57). This unique situation was used to study the development of cefotaxime resistance and to understand the contribution of genes known to play a role in this phenotype, *pbp2x*, *pbp1a*, *murM*, and *ciaH*, by analyzing the physiological and biochemical consequences in mutants containing various combinations of these genes.

## RESULTS

### β-Lactam resistance determinants in S. pneumoniae Hu17 and Hu15.

**FIG 1:**
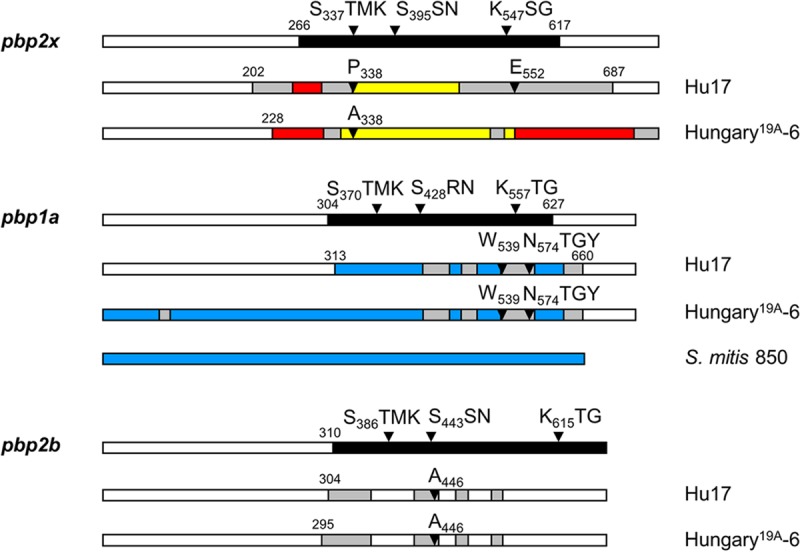
Mosaic PBPs of S. pneumoniae Hu17 and Hungary^19A^-6. Mosaic gene structures were deduced by comparison of the reference PBP2x sequences of strains S. pneumoniae R6 (white) and S. mitis M3 (red), NCTC10712 (yellow), and 850 (blue). Highly similar sequences (<5% difference) are shown in the same color; gray areas are divergent sequences of unknown origin. The numbers indicate the codons defining the sequence blocks that diverge from the *pbp2x*_R6_ nucleotide sequence by >15%. The domain structure and active-site boxes are indicated on top; the black area represents the transpeptidase domain. Mutations potentially relevant for the resistance phenotype are indicated.

To identify penicillin resistance determinants, two strains of ST226 were chosen for genome sequencing: Hu17, which is one of the strains with the highest levels of penicillin and cefotaxime resistance held in the Kaiserslautern strain (KL) collection, and penicillin-sensitive strain Hu15 (60). Genes encoding the resistance determinants PBP2x, PBP2b, PBP1a, and MurM were analyzed in detail. All three PBP genes of Hu17 display a mosaic structure very similar to that of the PBP genes of the Hungary^19A^-6 genome (GenBank accession no. CP000936.1) (Fig. 1; see also Fig. S1 in the supplemental material). They contain one sequence block highly divergent from the sequence of S. pneumoniae R6 that covers the transpeptidase domain (*pbp2x* of strain Hu17 [*pbp2x*_Hu17_] and *pbp1a* of strain Hu17 [*pbp1a*_Hu17_]) or four smaller divergent sequence blocks in the transpeptidase domain (*pbp2b* of strain Hu17 [*pbp2b*_Hu17_]) (Fig. 1 and S1). The mosaic sequence block of *pbp2x*_Hu17_ contains regions highly similar (<5% divergence at the nucleotide level) to the sequence of *pbp2x* of S. mitis strains M3 and NCTC10712, which is common among the highly variable *pbp2x* sequences of serotype 19A isolates from Hungary (58). In the deduced protein sequence, the two mutations T_338_P and Q_552_E, close to the active site Ser_337_ and the K_547_SG box, respectively, are noteworthy. The combination of both mutations is rare and most likely contributes to the high penicillin resistance level of Hu17. E_552_ is selectable with cefotaxime (19). Whereas A_338_ is present in mosaic *pbp2x* of most PRSP isolates, P_338_ is uncommon and leads to higher levels of β-lactam resistance compared to those obtained with A_338_ (20).

The diverse block of *pbp1a*_Hu17_ contains regions highly similar to the *pbp1*a sequence of S. mitis 850 (GenBank accession no. JUQO01000231) (61) interspersed with smaller regions of unknown origin (Fig. 1 and S1). PBP1a_Hu17_ contains the mutation L_539_W and the alteration of four consecutive residues, T_574_SQF to NTGY, implicated in resistance (62).

The PBP2b_Hu17_ gene of Hu17 has four divergent sequence blocks within the transpeptidase domain, including the mutation T_446_A, important for penicillin resistance (17). The mosaic blocks of *pbp2b* are not related to any *pbp2b* sequence of S. mitis in GenBank, except for a small region between codons 309 and 357 which is similar to the *pbp2b* sequences of S. mitis strains DD26 (GenBank accession no. LQOD00000000.1) (39) and SK1080 (GenBank accession no. AFQV01000033).

The *murM* gene of strain Hu17 (*murM*_Hu17_) is identical to the *murM* gene of S. pneumoniae Hungary^19A^-6 and displays a mosaic structure compared to S. pneumoniae R6. Its sequence diverges from that of the *murM* of strain R6 (*murM*_R6_) by 17%, resulting in 74 amino acid (aa) changes. Curiously, the *murM* allele is almost identical to the *murM* allele of penicillin-sensitive S. mitis strain SK616 (GenBank accession no. NZ_AICR00000000) (14), which differs by only 17 nucleotides (nt) (or 8 aa) from the *murM*_Hu17_ sequence (Fig. S2) and by 12 nt (or 5 aa) from the *murM* sequence of S. pseudopneumoniae 294 (GenBank accession no. JVMO01000033) (61), which is of unknown antibiotic susceptibility. This demonstrates that *murM* has also been acquired from S. mitis, similar to mosaic blocks in the PBP genes of PRSP isolates.

In addition, Hu17 contains the unique *ciaH232* allele, resulting in the mutation N_78_D within the sensor domain; *ciaH232* is slightly less effective than the CiaH wild type in mediating CiaR-dependent transcription in the R6 background (36). The *ciaH232* allele does not confer β-lactam resistance in the R6 background, but since other *ciaH* alleles from laboratory mutants and clinical isolates mediate this phenotype (see reference 36 and references within), it was included in this study.

In penicillin-sensitive strain Hu15, the sequences of the three PBP genes are almost identical to those in S. pneumoniae R6 and do not contain mosaic blocks (Fig. S1). The *pbp2x* gene of Hu15 differs from that of R6 by 9 nt, resulting in only 1 aa change, I_175_V, in the N-terminal domain, and *pbp1a* differs by 9 nt (PBP1a) and 3 aa changes (A_124_T, A_388_D, and D_533_E), which are also present in *pbp1a*_Hu17_, while *pbp2b* differs by 3 nt and no amino acid changes. Surprisingly, both *murM*_Hu17_ and *ciaH232* are present in Hu15, suggesting that these genes do not contribute to resistance by themselves, i.e., in the absence of mosaic PBPs. Furthermore, this clearly documents that they were present in this particular clone prior to the introduction of PBP genes, the major β-lactam resistance determinants.

### Experimental outline.

**FIG 2:**
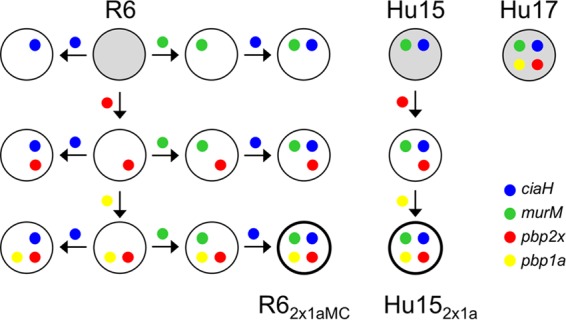
Schematic representation of transformation steps. The genes of PBP2x, PBP1a, MurM, and CiaH present in penicillin-resistant strain Hu17 (top right) were introduced individually or in various combinations into sensitive nonencapsulated laboratory strain S. pneumoniae R6 or into clinical isolate Hu15, as indicated by the colors. The direction of gene transfer is indicated by arrows. Gray circles represent recipient strains R6 and Hu15 and donor strain Hu17. Transformants containing all four genes are identified at the bottom and named according to the order of the transformation steps.

In this analysis, we focused on the development of cefotaxime resistance. Since PBP2b is not a target of this particular antibiotic and thus does not contribute to cefotaxime resistance, it was not included in the experiments (21). The genes encoding PBP2x, PBP1a, MurM, and CiaH of resistant strain Hu17 were introduced individually or in various combinations into nonencapsulated laboratory strain R6 (Fig. 2) to see how they affect the resistance level, as described below. In addition, Hu15 was used as the recipient of *pbp2x*_Hu17_ and *pbp1a*_Hu17_ to see whether they suffice to confer the cefotaxime resistance level of Hu17. Unlike most clinical isolates, including the resistant members of ST226, Hu15 is readily transformable under standard laboratory conditions and can be used in transformation experiments. Transformants were named according to the transferred genes, as shown in Fig. 2.

### β-Lactam resistance mediated by *pbp2x*_Hu17_ and *pbp1a*_Hu17_.

Transformations were carried out with PCR-amplified PBP_Hu17_ genes flanked by R6 or Hu15 sequences, as described in the supplemental material, to ensure that only *pbp2x*_Hu17_ or *pbp1a*_Hu17_ and not the flanking genes were introduced into the recipient strain. Cefotaxime was used as the selective antibiotic and was used at concentrations slightly above the MIC values for the recipient strains (for Hu15, 0.019 μg/ml; for R6, 0.018 μg/ml). Between 12 and 20 transformants were analyzed after incubation with BocillinFL, followed by sodium dodecyl sulfate (SDS)-polyacrylamide gel electrophoresis (PAGE) and fluorography, to identify transformants with low-affinity PBPs, and one such transformant was used in subsequent experiments.

**FIG 3:**
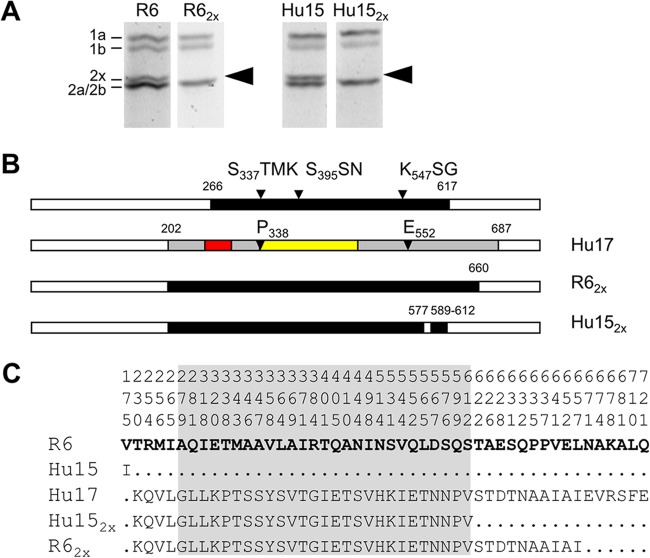
PBP2x variants obtained in transformation experiments. (A) PBP profiles of transformants containing mosaic PBP2x. PBPs were visualized after incubation with BocillinFL followed by separation by SDS-PAGE and fluorography. Recipient strains S. pneumoniae R6 and Hu15 were included for comparison, as indicated on top. The positions of the PBPs are marked on the left side. Arrowheads, low-affinity PBP2x. (B) Schematic representation of *pbp2x* in transformants R6_2x_ and Hu15_2x_ compared to strains R6 (white sequence blocks) and Hu17 (black sequence blocks). (Top row) The transpeptidase domain is shown in black, and the active-site motifs are indicated. (C) Deduced peptide sequences of PBP2x of the transformants compared to the peptide sequences of PBP2x of the penicillin-sensitive strain S. pneumoniae R6 and the donor PBP2x of Hu17. The first three lines indicate the amino acid positions. Only the residues at positions with altered residues are shown; residues identical to those in strain R6 (bold letters) are indicated by dots. The transpeptidase domain is shaded gray.

**FIG 4:**
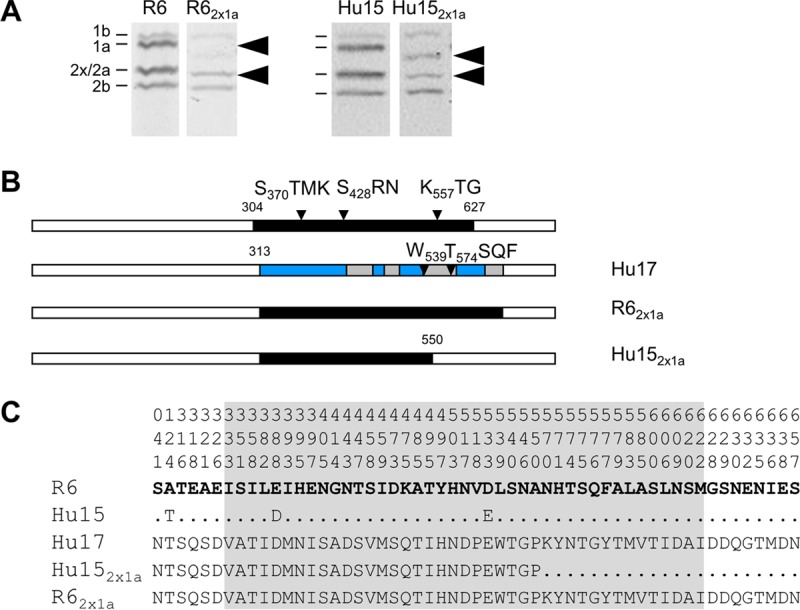
PBP1a variants obtained in transformation experiments. (A) PBP profiles of transformants containing mosaic PBP1a. PBPs were visualized after incubation with BocillinFL followed by separation by SDS-PAGE and fluorography. Recipient strains S. pneumoniae R6 and Hu15 were included for comparison, as indicated on top. The positions of the PBPs are marked on the left side. Arrowheads, low-affinity PBP2x and PBP1a. (B) Schematic representation of *pbp1a* in transformants compared to strains R6 (white sequence blocks) and Hu17 (black sequence blocks). (Top row) The transpeptidase domain is shown in black, and the active-site motifs are indicated. (C) Deduced peptide sequences of PBP1a of the transformants compared to the peptide sequences of PBP1a of the penicillin-sensitive strain S. pneumoniae R6 and the donor PBP1a of Hu17. The first three lines indicate the amino acid positions. Only the residues at positions with altered residues are shown; residues identical to those in strain R6 (bold letters) are indicated by dots. The transpeptidase domain is shaded gray.

**FIG 5:**
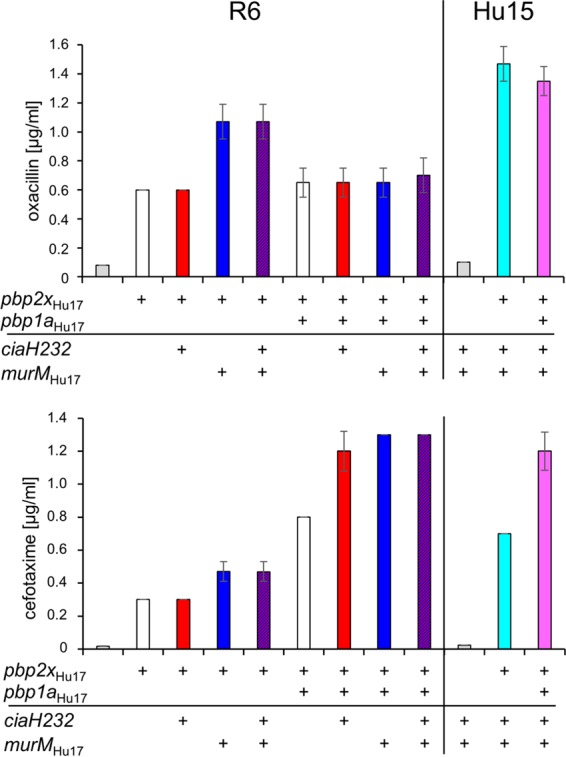
Resistance patterns of various S. pneumoniae transformants. The two β-lactams oxacillin and cefotaxime were used. Mean values from at least three independent experiments are shown. Bars indicate standard deviations. The MIC values of oxacillin and cefotaxime for the donor strain Hu17 were 30 μg/ml and 1.6 μg/ml, respectively.

First, the *pbp2x*_Hu17_ gene was introduced into strain Hu15. The transformant, Hu15_2x_, contained almost the entire transpeptidase domain of *pbp2x*_Hu17_, including the deduced mutations P_338_ and E_552_ (Fig. 3). Subsequent transfer of *pbp1a*_Hu17_ resulted in Hu15_2x1a_, where *pbp1a*_Hu17_ was present up to codon 550, a sequence which includes the deduced mutation W_539_ (Fig. 4). The MIC values of oxacillin and cefotaxime for Hu15_2x_ were 1.5 and 0.7 μg/ml, respectively (Fig. 5). The cefotaxime MIC for Hu15_2x1a_ was 1.2 μg/ml, which is significantly higher than the MIC for Hu15_2x_ and close to the MIC for the parental strain, Hu17 (1.6 μg/ml) (Fig. 5). The slight difference might be attributed to the fact that the entire *pbp1a*_Hu17_ sequence, including codons 574 to 577, is not present in Hu15_2x1a_. In contrast, no increase in the oxacillin MIC was mediated by *pbp1a*_Hu17_; rather, a slight but reproducible decrease in the oxacillin MIC was observed (Fig. 5). This suggests that introduction of *pbp1a*_Hu17_ into this particular genetic background requires cefotaxime selection and that the increased oxacillin resistance is related to other genes not included in the transformation experiments. Nevertheless, these data document that the four genes *ciaH232*, *murM*_Hu17_, *pbp2x*_Hu17_, and *pbp1a*_Hu17_ suffice to mediate high levels of resistance to cefotaxime in Hu15.

The transformant R6_2x_ contained the *pbp2x*_Hu17_ sequence up to codon 660, resulting in a considerable increase in cefotaxime and oxacillin MICs (0.3 μg/ml and 0.6 μg/ml, respectively) compared to those for the parental R6 strain (0.018 μg/ml and 0.08 μg/ml, respectively), as expected (Fig. 3 and 5). Successive transformation of the entire *pbp1a*_Hu17_ sequence increased the resistance levels even more, especially for cefotaxime (0.8 μg/ml); the increase in the oxacillin MIC was less pronounced (0.65 μg/ml) (Fig. 4 and 5). However, these values are below the MICs for Hu15_2x1a_, clearly documenting that at least *murM*_Hu17_ contributes to β-lactam resistance and *ciaH232* potentially contributes to β-lactam resistance.

### Effects of *murM*_Hu17_ and *ciaH232* on β-lactam resistance and cell morphology in S. pneumoniae R6.

**FIG 6:**
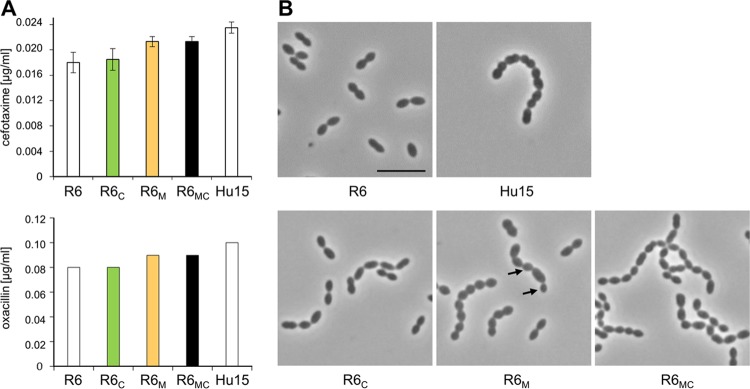
Cell morphology and resistance pattern of S. pneumoniae R6 transformants carrying the *murM* and/or *ciaH* gene from strain Hu17. (A) The MIC values for the transformants are indicated as colored bars, and those for control strains R6 and Hu15 are indicated as white bars. The two β-lactams cefotaxime and oxacillin were used. Mean values from at least three independent experiments are shown. Bars indicate standard deviations. (B) Representative phase-contrast micrographs are shown. The strains were grown in C+Y medium. Arrows, altered cell morphology. Bar, 5 μm.

Although S. pneumoniae strains Hu15 and R6 are penicillin susceptible, they differ slightly in their MIC values for oxacillin and cefotaxime (Fig. 6). To assess the contribution of *murM*_Hu17_ and *ciaH232* to β-lactam resistance, the genes were introduced individually or in combination into S. pneumoniae R6 using the Janus counterselectable procedure (63). This avoids selection with β-lactam antibiotics, and the *rpsL41* mutation, which is required for such constructs, has no effect on CiaRH-mediated regulation or β-lactam susceptibility (36).

The transformant R6 *murM*_Hu17_ (R6_M_) grew with a longer generation time of 68 min and a shorter stationary phase (approximately 2 h) compared to those for R6 and R6 *ciaH232* (R6_C_) (51 min and approximately 4 h, respectively) and was only slightly but reproducibly less susceptible to oxacillin and cefotaxime (Fig. 6A). However, the MIC values of cefotaxime and oxacillin for R6_M_ were still below those for Hu15. In addition, the cell morphology of R6_M_ was grossly altered. The cells were round, similar to Hu15 cells, and distinct from the oval-shaped cells of the parental R6 strain, and 50% of the cells grew in chains containing occasionally enlarged or smaller cells (Fig. 6B). In contrast, R6_C_ grew with the same generation time as the parental R6 strain (not shown), and its cell morphology was not affected (Fig. 6B). The visual impression was confirmed by precise determination of the cell dimensions (see Materials and Methods). In fact, the length and width of R6_C_ cells (0.96 μm ± 0.04 and 0.83 μm ± 0.03, respectively; *n* = 206) were identical to the values obtained for R6 cells (0.95 μm ± 0.04 and 0.80 μm ± 0.03, respectively; *n* = 220).

Introduction of *ciaH232* into R6_M_ (R6_MC_) had no effect on MIC values but remarkably affected cell morphology. R6_MC_ cells were more oval shaped than R6_M_ cells, and the morphology of R6_MC_ cells was similar to that of cells of the parental R6 strain, whereas chain formation was more pronounced in R6_MC_ cells than parental R6 cells (Fig. 6B). In other words, the morphological defects caused by *murM*_Hu17_ were almost reversed by *ciaH232*, while cells still grew in chains, suggesting that the CiaRH system somehow responds to the altered cell wall biochemistry mediated by *murM*_Hu17_.

### Effect of *ciaH232* and *murM*_Hu17_ on β-lactam resistance in S. pneumoniae R6_2x_ and R6_2x1a_.

In further experiments, *ciaH232* and *murM*_HU17_ were transferred into strains R6_2x_ and an R6 strain which carried *pbp2x*_Hu17_ and *pbp1a*_Hu17_ (R6_2x1a_) individually or in combination to study their impact on resistance in the presence of *pbp2x*_Hu17_ alone or together with *pbp1a*_Hu17_. The introduction of *murM*_Hu17_ into R6_2x_ resulted in strain R6_2xM_ and conferred increased cefotaxime and oxacillin MICs (Fig. 5). This demonstrates for the first time that *pbp2x*_Hu17_ alone suffices as a genetic background to reveal that a mosaic *murM* contributes substantially to β-lactam resistance. In contrast, transfer of *ciaH232* into strain R6_2x_ (R6_2xC_) or R6_2xM_ (R6_2xMC_) had no effect on MIC values (Fig. 5).

When transformed into R6_2x1a_, *murM*_Hu17_ resulted in a further increase in the level of cefotaxime resistance (1.3 μg/ml compared to 0.8 μg/ml for R6_2x1a_). Surprisingly, transfer of *ciaH232* into R6_2x1a_ also resulted in a significant increase in the cefotaxime MIC value to 1.2 μg/ml (Fig. 5). Resistance associated with *ciaH* was first observed in laboratory mutants and led to hyperactivation of the CiaR regulon (64). Since *ciaH232* does not mediate resistance by itself in the R6 background (36), this finding suggests that *ciaH232* can indeed hyperactivate the CiaR regulon but only in the presence of both *pbp1a*_Hu17_ and *pbp2x*_Hu17_ (R6_2x1aC_) and not in the sole presence of *pbp2x*_Hu17_ (R6_2xC_). This is the first time that a phenotype conferred by a *ciaH* allele appears to be associated with an altered PBP1a. Moreover, this effect was seen only in the absence of *murM*_Hu17_; i.e., transfer of *ciaH232* into R6_2x1aM_ did not result in a further increase in the level of β-lactam resistance (Fig. 5).

Curiously, the oxacillin resistance level of all R6 transformants containing *pbp1a*_Hu17_ was clearly below the MIC for R6_2xM_ or R6_2xMC_ mediated by *murM*_Hu17_, as if the presence of *pbp1a*_Hu17_ somehow suppresses the *murM*_Hu17_-mediated resistance (Fig. 5). It should also be noted that the oxacillin resistance level of Hu15_2x1a_ was almost 2-fold higher than that of R6_2x1aMC_. This strongly suggests the presence of unknown features that feed into the resistance phenotype of the clone Hungary^19A^-6.

### Activity of CiaR-controlled promoters in the presence of resistance determinants.

Various *ciaH* alleles identified in laboratory mutants contribute to resistance and result in a hyperactive CiaRH system, whereas these features were less pronounced with three distinct CiaH alleles of clinical isolates or even absent in the case of *ciaH232* (36). However, the effect of *ciaH232* on cefotaxime resistance when it was introduced into R6_2x1a_ as described above suggested that the CiaRH system is also hyperactive under these conditions. Therefore, the expression of two CiaR-dependent genes encoding the serine protease HtrA and a secreted protein of unknown function, spr0931, was tested in different R6 derivatives using *lacZ* reporter assays (35, 65). The activities of both the P_*htrA*_ and P_spr0931_ promoters are strongly dependent on CiaR, and there is no evidence that they are controlled by other regulators (35, 50). Each of the promoters was cloned in front of a promoterless Escherichia coli *lacZ* gene (65) and integrated into the S. pneumoniae R6 strain and its derivatives. We selected for further experiments strains which carried *ciaH232* in combination with altered *pbp2x*_Hu17_ (R6_2x_), strains which carried *pbp2x*_Hu17_ and *pbp1a*_Hu17_ (R6_2x1a_), strains which did not carry *pbp2x*_Hu17_ (R6_C_), and the parental R6 strain as a control.

**FIG 7:**
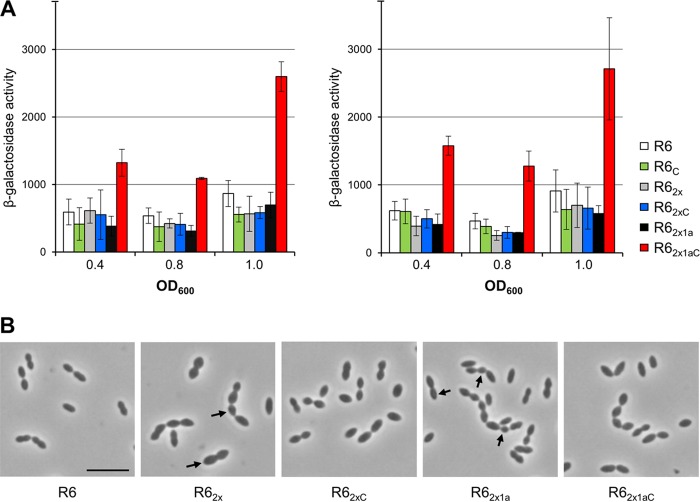
β-Galactosidase activities expressed from CiaR-regulated promoters and cell morphology of S. pneumoniae R6 derivatives carrying the *ciaH232* allele. (A) Strains were grown in C+Y medium. β-Galactosidase activities were determined at three different time points and are given in nanomoles of nitrophenol produced per minute and milligram of protein. Mean values and standard deviations from three independent experiments are presented. The activities of the CiaR-dependent promoters in the R6_2x1aC_ strain were significantly different (*P* < 0.05) from those of the promoters in the other strains. The promoters and relevant genetic markers are indicated. (B) Phase-contrast images taken at the mid-exponential growth phase. The strains were grown in C+Y medium. Arrows, altered cell morphology. Bar, 5 μm.

β-Galactosidase activities were measured in cells grown in complex C medium (66) supplemented with 0.1% yeast extract (C+Y medium) at two different time points during exponential growth (when the optical density at 600 nm [OD_600_] was 0.4 and 0.8) and at the onset of the stationary phase. As shown in Fig. 7A, the levels of transcription from both promoters P_*htrA*_ and P_spr1149_ were similar in all strains except R6_2x1aC_, where promoter activities increased more than 3-fold and were even higher at the onset of stationary phase. These results confirmed that the CiaRH system is hyperactive in the presence of *pbp1a*_Hu17_ in combination with *pbp2x*_Hu17_.

### Effect of CiaH232 and mosaic PBP2x_Hu17_/PBP1a_Hu17_ on morphology and peptidoglycan composition.

The hyperactivation of CiaR observed in strain R6_2x1aC_ indicates that the sensor kinase CiaH232 responds to some signal mediated by PBP1a_Hu17_. Since PBP1a acts as a transglycosylase/transpeptidase during PG synthesis, it is possible that R6_2x1aC_ produces an altered PG. Therefore, transformants containing *pbp2x*_Hu17_ or both *pbp2x*_Hu17_ and *pbp1a*_Hu17_ with or without *ciaH232* were examined microscopically, and the PG composition of the strains was investigated.

The presence of *pbp2x*_Hu17_ in R6_2x_ already resulted in cells that were enlarged and more rounded compared to cells of the parental R6 strain, an effect which was at least partially reverted by *ciaH232* in R6_2xC_ (Fig. 7B). The additional presence of *pbp1a*_Hu17_ (R6_2x1a_) resulted in cells with more pointed ends, and chain formation and smaller cells were frequently observed, suggesting some defects related to cell division and separation. Again, the subsequent introduction of *ciaH232* resulted in cells that appeared more normal in size and form (Fig. 7B). These results suggest that the function of the products of both *pbp2x*_Hu17_ and *pbp1a*_Hu17_ differs from that of the respective R6 proteins and, thus, that these products affect cell morphology, whereas *ciaH232* counteracts this defect.

The peptidoglycan was isolated from the transformants, and the compositions of the muropeptides were analyzed as described previously (24). Detailed analysis of the PG of S. pneumoniae by reversed-phase high-performance liquid chromatography (HPLC) revealed 50 different muropeptide structures, including those carrying modifications such as deacetylation of Glc*N*Ac residues and *O*-acetylation of Mur*N*Ac residues, the presence of iGln instead of Glu, and Gly at position 5 of the stem peptide (24). In the present study, 31 peaks which included all major muropeptides, accounting for 68 to 72% of known muropeptides, were used for the final analysis (Table S1).

All mutants retained an overall muropeptide composition similar to that of parental strain R6 (Tables 1 and S1). Small changes (differences of up to 15% from the values for R6) were seen in the total number of monomers, dimers, and trimers between the PG of the strains. However, changes became evident when individual muropeptide classes were analyzed in detail (Table 1). Remarkably, the presence of *ciaH232* already resulted in a slight increase in the amount of pentapeptides by 18% in R6_C_ compared to R6 and a relative increase in the ratio of indirectly cross-linked dimers versus directly cross-linked dimers from 1.19 in R6 to 1.61 in R6_C_ (a 35% increase compared to that in R6), in parallel with a higher ratio of branched peptides versus linear peptides (from 0.44 to 0.51, or 16%). Moreover, the proportion of muropeptides with deacetylated Glc*N*Ac increased only slightly from 20.7% (R6) to 22% (Table 1). This suggests that the CiaH232 allele in not neutral for the cell and may alter the cell wall precursor metabolism to be compatible with PBPs that prefer branched substrates.

**TABLE 1:** Cell wall muropeptide compositions of S. pneumoniae R6 and mutant strains

Muropeptide	Relative peak area (%) or ratio in strain^*a*^:					
	R6	R6_C_	R6_2x_	R6_2xC_	R6_2x1a_	R6_2x1aC_
Monomers	56.8 ± 0.8	56.9 ± 1.2	60.1 ± 0.1	61.0 ± 1.7	60.5 ± 0.2	59.4 ± 1.1
Dimers	39.0 ± 0.8	38.6 ± 1.6	35.2 ± 0.1	34.4 ± 1.6	34.9 ± 0.1	35.7 ± 1.2
Trimers	4.2 ± 0.0	4.5 ± 0.3	4.7 ± 0.3	4.7 ± 0.1	4.6 ± 0.2	4.9 ± 0.1
Tripeptides	65.7 ± 0.8	65.1 ± 0.3	64.4 ± 1.2	64.6 ± 0.8	64.3 ± 0.6	64.4 ± 1.5
Tetrapeptides	26.1 ± 0.9	26.5 ± 1.2	25.2 ± 0.6	25.1 ± 0.2	26.3 ± 1	26.1 ± 0.5
Pentapeptides	6.0 ± 1.4	7.1 ± 1.4	**9.4 ± 1.7**	**9.3 ± 0.6**	**8.7 ± 1.6**	**8.8 ± 1.0**
Peptides in cross-links	43.2 ± 0.8	43.1 ± 1.2	39.9 ± 0.2	39.0 ± 1.7	39.5 ± 0.2	40.6 ± 1.1
Directly cross-linked dimers	17.8 ± 1.0	14.8 ± 0.8	13.2 ± 0.6	13.2 ± 0.9	**11.8 ± 0.5**	12.5 ± 1.0
Indirectly cross-linked dimers	21.2 ± 1.8	23.8 ± 0.8	22.0 ± 0.5	21.2 ± 0.7	23.2 ± 0.6	23.2 ± 0.2
Ratio of indirectly cross-linked dimers/directly cross-linked dimers	1.19	**1.61**	**1.7**	**1.61**	**1.97**	**1.86**
Linear peptides	63.9 ± 2.4	60.7 ± 1.3	59.5 ± 2.1	59.5 ± 1.0	57.4 ± 0.8	57.5 ± 0.6
Branched peptides^*b*^	27.9 ± 1.9	30.9 ± 1.1	32.2 ± 2.1	31.7 ± 0.7	32.6 ± 0.2	33.2 ± 0.5
Ratio of branched peptides/linear peptides	0.44	0.51	0.54	0.53	**0.57**	**0.58**
Deacetylation	20.7 ± 0.1	21.9 ± 0.1	23.0 ± 1.9	23.3 ± 0.7	25.0 ± 3.2	24.0 ± 0.2

a The values are means ± variations for two independent PG preparations. Laura software (Lab Logic Systems Ltd) was used for peak area quantification. The relative peak areas were estimated as the percentage of all known peaks. Underlined values were considered to be significantly different by more than 15% from the value for strain R6; bold underlined values were significantly different by more than 30% from the value for R6.

b Muropeptides with an SA or AA branch.

Major differences in muropeptide compositions were observed in constructs containing the mosaic PBPs (Table 1). In all four mutants, the percentage of directly cross-linked dimeric peptides decreased substantially, while the percentage of indirect cross-links slightly increased, resulting in an overall slightly reduced amount of dimers. The percentage of pentapeptides, muropeptides with deacetylated Glc*N*Ac, and branched peptides increased further in all strains containing mosaic PBP genes. The additional presence of *ciaH232* (R6_2xC_) had no effect compared to the effect on R6_2x_. In R6_2x1a_, indirectly cross-linked dimers were present almost twice as often as directly cross-linked peptides, and the proportion of muropeptides with deacetylated Glc*N*Ac increased slightly to 25%. In other words, the presence of PBP2x_Hu17_ and PBP1a_Hu17_ as well resulted in a different PG composition, indicating that their alterations apparently affected their substrate specificity, and surprisingly, *ciaH232* also had an impact on the muropeptide profile.

Nevertheless, our PG analysis shows the remarkable robustness of PG synthesis in S. pneumoniae that allows the cell to produce an almost normal PG with an altered regulatory CiaRH system and two mosaic synthases, PBP2x and PBP1a, which lead in their original strain background to a different, more branched PG. Yet this specificity of the PBPs does cause structural changes in the PG, including a decrease in direct cross-links and an increase in pentapeptides and deacetylated muropeptides, which may contribute to the morphological defects observed.

## DISCUSSION

The results presented here reveal new aspects of resistance development in clinical isolates of S. pneumoniae by analyzing the physiological consequences associated with the transfer of resistance determinants into laboratory strain R6. The high-level penicillin-resistant clone Hungary^19A^-6 contains four genes involved in cefotaxime resistance: the well-known penicillin target enzymes PBP2x and PBP1a and the non-PBP genes *murM* and *ciaH232*. We used the unique situation of penicillin-sensitive strain Hu15, which is of the same sequence type (ST226) as strain Hu17 and which contains both an altered *murM* and the *ciaH232* allele, to show that these two genes were already present before the introduction of altered PBP genes, i.e., most likely long before the extensive use of β-lactam antibiotics triggered the evolution of PRSP. In the following sections we discuss features associated with these four genes.

### *pbp2x* and *pbp1a*.

Both *pbp2x*_Hu17_ and *pbp1a*_Hu17_ represent rare mosaic variants similar to alleles present in other Hungary isolates of ST226 (57, 58) (Fig. 1; see also Fig. S1 in the supplemental material). Both PBPs contain mutations close to active-site motifs. P_338_ and E_552_ in PBP2x_Hu17_ have been shown experimentally to play a role in resistance development (19, 20). Also, the change Q_599_P, a mutation at a site which is affected in high-level cefotaxime-resistant strain S. mitis B6 (W_599_) (67), might contribute to the high level of cefotaxime resistance of strain Hu17. There are other alterations in PBP2x_Hu17_ frequently found in PBP2x of PRSP strains: A_369_V, I_371_T, N_444_S, and S_531_K (15). The alterations A_279_G, I_318_L, E_320_K, T_401_I, Q_405_E, A_410_T, V_544_I, L_565_T, and S_612_V have not yet been described, and it remains to be clarified experimentally whether these changes contribute to resistance. All other alterations occur in the parental sequence blocks related to S. mitis strains M3 and NCTC10712.

PBP1a_Hu17_ clearly contributes to cefotaxime resistance but only slightly contributes to oxacillin resistance (58), indicating that its mutations primarily affect the interaction with cefotaxime. Similar results were obtained when mosaic PBP genes of the clone Spain^23F^-1 were transferred into the R6 strain (20). Curiously, the oxacillin resistance level of all R6 transformants containing *pbp1a*_Hu17_ was clearly below the MIC of R6_2xM_ or R6_2xMC_ mediated by *murM*_Hu17_, as if the presence of *pbp1a*_Hu17_ somehow suppresses the *murM*_Hu17_-mediated resistance (Fig. 5). The oxacillin resistance level of Hu15_2x1a_ was almost 2-fold higher than that of R6_2x1aMC_, strongly suggesting the presence of unknown features that feed into the resistance phenotype of the ST226 strains.

The A_124_T and E_388_D mutations in PBP1a lead to strong suppression of the essentiality of *mreCD*. PBP1a and MreCD are components proposed to be part of the same protein complex involved in peripheral PG synthesis (68). These changes are not present in strain R6 but are present in strains Hu15 and Hu17. It should also be noted that *mraY*, located downstream of *pbp2x*, is highly altered in strains Hu17 and Hu15. MraY is an essential phospho-*N*-acetylmuramoyl pentapeptide transferase in PG synthesis. These gene products could have an impact on cell morphology and PG composition in the Hungary^19A^-6 clone and might affect PBP function indirectly.

Although the overall PG composition appeared to be similar in all constructs analyzed, differences which affected the ratio of individual muropeptides, the substrate, and the product of PBPs were revealed. These differences could be due to modifications in the availability of the different substrates or to endopeptidases hydrolyzing the PG cross-links. However, we believe it to be more likely that the PBPs themselves were responsible for these changes. All R6 derivatives containing PBP2x_Hu17_ or both PBP2x_Hu17_ and PBP1a_Hu17_ showed an increased ratio of indirectly over directly cross-linked dimers, ranging from an increase compared to the ratio in R6 of 43% in R6_2x_ to 65% in R6_2x1a_ (Table 1). This implies that both PBPs have an altered substrate specificity and prefer branched over linear peptides as the substrate, as has been previously suggested (27). We have now demonstrated for the first time experimentally an impact of individual mosaic PBPs on PG composition. Enzymatic analyses of purified PBPs are required to reveal in detail the differences in the kinetic properties of PBPs between resistant strains and sensitive strains.

The R6 strain used in the present study has mostly linear peptides but a higher percentage of branched peptides and indirect cross-links than its progenitor strains, D39 and R36A (24, 26, 28, 69). Therefore, it is possible that mosaic PBPs can affect the PG composition more prominently in different genetic backgrounds.

There were other differences in muropeptide composition that cannot be explained by the sole action of PBP2x and PBP1a. The increase in pentapeptides in strains with the mosaic *pbp2x* gene suggests either that the trimming of nascent pentapeptides to tetra- and tripeptides by PBP3 (70) and LdcB (DacB) (71) is affected or that the overall synthesis of PG is increased to an extent that pentapeptides are not trimmed fast enough. Notably, the cells of R6_2x_ and R6_2x1a_ exhibited morphological defects similar to those of cells of a *dacB* deletion strain (72). Finally, strains with mosaic PBP genes contained more deacetylated muropeptides; whether this was due to an increased activity of the PG deacetylase PgdA remains to be clarified (73).

### Effects mediated by *murM*_Hu17_.

The presence of an altered MurM gene in PRSP isolates of serotype 19A strains from Hungary and its contribution to penicillin resistance in the presence of altered PBP genes have previously been described for strains Hun663 (30, 74) and 3191 (32). In contrast, many other PRSP clones do not contain mosaic MurM genes, including the clone Taiwan^19F^-14 and serotype 19A switch variants thereof (75) and the clonal complex Spain^23F^-1. The mosaic *murM* was present not only in penicillin-resistant strain Hu17, as expected, but also, as shown here for the first time, in penicillin-sensitive strain Hu15, which does not contain mosaic PBP genes. The gene *murM*_Hu17_ is highly related to *murM* of the penicillin-sensitive S. mitis strain SK616 (14), differing in only 5 aa, strongly suggesting that it was acquired from S. mitis. This is not surprising, given the fact that Hungary^19A^-6 had acquired the largest proportion of genes (8.2%) from S. mitis in a comparative genomic analysis of 35 Streptococcus species genomes (76).

Two amino acid changes in MurM_Hu17_ are present in the region between residues 244 and 274 proposed to be important for the contribution to penicillin resistance (77): K_266_ and P_267_. When introduced into S. pneumoniae R6, *murM*_Hu17_ contributed only marginally to β-lactam resistance (Fig. 5); similarly, only a small increase in β-lactam susceptibility has been noted in R6 derivatives with a deleted *murM* (31). Previous analyses have studied the effect of *murM* only in the presence of multiple mosaic PBP genes (30, 32). As shown here, MurM_Hu17_ already caused changes in morphology and growth in the R6 background (Fig. 6), and a substantial increase in β-lactam resistance was observed when it was introduced into R6_2x_; i.e., MurM_Hu17_ does not require PBP1a to mediate a clear resistance effect (Fig. 5). Thus, it could well be that the presence of the mosaic *murM* is not related to a selective advantage during exposure to β-lactams but that it has been circulating in this clone long before the transfer of PBP sequences. In agreement with this, the MurM gene in strains Hu15, Hu17, and Hungary^19A^-6, including the upstream gene encoding a putative tributyrin esterase, is completely conserved, whereas PBP genes and flanking regions (*pbp2x* and *mraY*, *clpL*; *recU* and *pbp1a*; *pbp2b* and *ddl*) are diverse, as has been described in other PRSP isolates (78). This scenario suggests that the mosaic PBPs, after all essential enzymes, must cope with MurM_Hu17_ with different enzymatic properties (29), resulting in the abundant presence of branched muropeptides (27), i.e., the substrates of PBPs. In other words, some mutations in the mosaic PBPs might be related to an altered substrate pool and not necessarily to β-lactam resistance in this particular genetic background.

### The allele *ciaH232*.

Almost no effects of *ciaH232* on β-lactam resistance or the expression of CiaR-regulated genes were observed when it was introduced into the R6 strain, in contrast to the findings for *ciaH* alleles from laboratory mutants (36). However, cells containing *ciaH232* reacted more strongly to the presence of acetate in the medium (79). In the present study, some new features of *ciaH232* that are associated with cell morphology, β-lactam resistance, and cell wall composition were revealed. The first example refers to the comparison of the transformants R6_M_ and R6_MC_, where R6_M_ cells grew with an aberrant morphology, whereas R6_MC_ cells were indistinguishable from those of the parental R6 strain (Fig. 5). Similarly, the morphological changes induced by PBP2x_Hu17_ and PBP1a_Hu17_ were compensated for by the presence of *ciaH232* (Fig. 7B). This suggests that CiaH232 apparently responds to alterations mediated directly or indirectly by the resistance determinants of Hu17 to ensure proper cell growth. Second, cefotaxime resistance was further increased when *ciaH232* was transformed into R6_2x1a_ but not into R6_2x_ (Fig. 6), and the CiaR-regulated genes *htrA* and spr0931 showed higher expression levels only in R6_2x1aC_ (Fig. 7A). The N_78_D mutation of CiaH232 is located in the sensor domain of the histidine protein kinase, in agreement with an altered signal recognition site outside the cell membrane, mediated by *murM*_Hu17_ and *pbp1a*_Hu17_ or *pbp2x*_Hu17_. Thus, it is possible that the CiaH mutation expressed by *ciaH232* is a response to the presence of *murM*_Hu17_ in the Hungary^19A^-6 strain.

These data were complemented by cell wall analysis, which revealed changes in the PG composition in R6 derivatives containing *pbp1a*_Hu17_ and/or *pbp2x*_Hu17_ (Tables 1 and S1); *murM*-mediated changes in PG composition have been reported before (30, 74). Curiously, enhanced expression of the CiaR-regulated genes was observed only in R6_2x1aC_ and not in R6_2xC_, similar to previous reports that showed that the wild-type *ciaH* allele did not affect *htrA* expression in the presence of PBP2x point mutations or another mosaic PBP2x from a clinical isolate (37). However, CiaR activation by CiaH from clinical isolates of S. pneumoniae was far less pronounced than that by CiaH from laboratory mutants (36). This is an indication that mutations in PBP2x and CiaH selected with β-lactams in laboratory mutants affect the function of both proteins differently and with a more detrimental outcome to the cells compared to the outcome resulting from mutated alleles in clinical isolates. Altogether this scenario adds another facet to the interplay between the CiaRH system and PBPs and differences in PBP mutations of laboratory mutants versus clinical isolates.

Most intriguing was the finding that the muropeptide composition was affected by *ciaH232* (R6_C_) in the absence of PBPs_Hu17_: an increase in the amount of pentapeptides by 18%, an increased proportion of indirectly cross-linked dimers (12%), and more muropeptides containing a deacetylated Glc*N*Ac (5.8%). The values are less than those seen for R6_2x_ and R6_2x1a_ but concern the same muropeptide classes. These data are in agreement with the assumption that the CiaRH system somehow controls the overall composition of the pneumococcal cell wall to ensure its integrity (20, 37).

### Concluding remarks.

**FIG 8:**
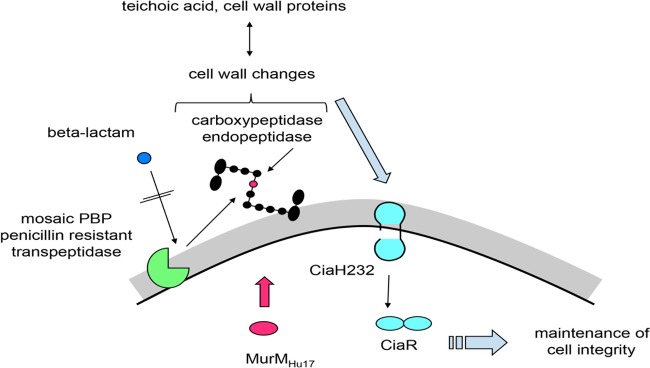
Schematic view of the interactions between MurM, PBPs, and the CiaRH system. Black, a branched muropeptide; red, l-Ala in the interpeptide bridge added by MurM_Hu17_; gray: cell wall; black line, cell membrane. See the text for details.

In the early 1990s, the penicillin resistance of pneumococci was considered to be entirely due to altered PBPs (80). The non-PBP components CiaH and MurM have since been recognized to be relevant players in resistance development in laboratory mutants and clinical isolates, respectively. The present analysis revealed that the CiaH232 allele of the S. pneumoniae clone Hungary^19A^-6 responds to the presence of MurM_Hu17_. The contribution to β-lactam resistance of these two genes in combination with mosaic PBP genes carrying mutations known to be relevant for resistance development became evident. The interplay between the cytoplasmic MurM involved in the biosynthesis of PG precursors, transpeptidases controlling the PG cross-linkage at the outer surface of the cell wall (PBP2x and PBP1a), and the sensor histidine protein kinase CiaH mediating signals from the outside to the response regulator CiaR is schematically shown in Fig. 8. Taken together, the data reveal a highly complex network that ensures the synthesis of a functional bacterial cell wall under antibiotic stress.

## MATERIALS AND METHODS

### Bacterial strains, plasmids, and growth conditions.

The bacterial strains used in this study are listed in Table 2. S. pneumoniae strains were grown at 37°C without aeration in complex C medium (66) supplemented with 0.1% yeast extract (C+Y medium) or in brain heart infusion (BHI; Roth) medium, and growth was followed by monitoring the optical density at 600 nm (OD_600_). Strains were grown on D-agar plates (81) supplemented with 3% defibrinated sheep blood.

**TABLE 2:** S. pneumoniae strains used in this study

Strain	Relevant characteristics	Reference(s) or source
R6^*a*^	Unencapsulated laboratory strain, β-lactam susceptible	85, 86
Hu15	Serotype 19A isolate from Hungary, β-lactam susceptible	57
Hu17	Serotype 19A isolate from Hungary, β-lactam resistant	57
CCCO*murM*::Janus	CCCO *murM*::Kan^r^ *rpsL^+^ rpsL41* Kan^r^ Str^r^	12
CCCO*murM*_Hu17_	CCCO *murM*_Hu17_ *rpsL41* Str^r^	12
RKL243	R6 *ciaH232 rpsL41* Str^r^	36
RKL248	RKL243 *bgaA*::*tet*(M)-P*_htrA_* Str^r^ Tet^r^	36
RKL249	RKL243 *bgaA*::*tet*(M)-P_spr0931_ Str^r^ Tet^r^	36
RKL161	R6 *ciaH*::Kan^r^ *rpsL^+^ rpsL41* Kan^r^ Str^s^	36
R6^strR^	R6 *rpsL41* Str^r^	This study
R6_M_	R6 *murM*_Hu17_ *rpsL41* Str^r^	This study
R6_MC_	R6_M_ *ciaH232* Str^r^	This study
R6_2x_^*a*^	R6 *pbp2x*_Hu17_	This study
R6_2xC_^*a*^	R6_2x_ *ciaH232 rpsL41* Str^r^	This study
R6_2xM_	R6_2x_ *murM*_Hu17_ *rpsL41* Str^r^	This study
R6_2xMC_	R6_2xM_ *ciaH232* Str^r^	This study
R6_2x1a_^a^	R6_2x_ *pbp1a*_Hu17_	This study
R6_2x1aM_	R6_2x1a_ *murM*_Hu17_ *rpsL41* Str^r^	This study
R6_2x1aC_^*a*^	R6_2x1a_ *ciaH232* Str^r^	This study
R6_2x1aMC_	R6_2x1aM_ *ciaH232* Str^r^	This study
Hu15_2x_	Hu15 *pbp2x*_Hu17_	This study
Hu15_2x1a_	Hu15_2x_ *pbp1a*_Hu17_	This study

a The promoter-probe plasmids carrying *htrA* or spr0931 promoter fragments (35) were integrated into the *bgaA* locus of R6 derivatives; therefore, the strains are deficient in endogenous β-galactosidase activity due to disruption of *bgaA*.

Promoter-probe plasmid pPP2 containing the *htrA* and spr0931 promoters and plasmid pPP2 have been described previously (35, 65). For cloning and propagation of plasmids, Escherichia coli strain DH5α was used as a host. E. coli strains were grown aerobically at 37°C either in LB medium or on LB agar plates (82). The growth of E. coli was followed by measuring the OD_600_ using a spectrophotometer.

### Microscopy and growth curves.

For physiological and morphological analysis, cells were inoculated in prewarmed C+Y or BHI medium and grown at 37°C without aeration. Cell growth was monitored by spectroscopy at OD_600_. At an OD_600_ of 0.7, cultures were diluted 1:20 in the respective prewarmed medium, and growth was followed throughout the growth cycle. For microscopic analysis, 5 μl of exponential growing cells at an OD_600_ of 0.7 was transferred to poly-l-lysine-coated slides and analyzed by phase-contrast microscopy using an Eclipse E600 (Nikon) microscope equipped with a 100× oil immersion objective (numerical aperture, 1.4). Photographs were taken with a DXM1200C camera (Nikon). Image analysis and determination of the cell size were carried out using Nikon Nis-Elements BR (version 3.2) imaging software. For physiological and morphological analyses, the cells from three separate cultures were analyzed, and at least two photographs of each culture were taken.

### Transformation procedure.

Transformation of the S. pneumoniae strains was carried out as described previously (37). When required, the growth media for S. pneumoniae were supplemented with the following antibiotics: 200 μg/ml kanamycin (Kan), 200 μg/ml streptomycin (Str), 3 μg/ml tetracycline (Tet), or 20 μg/ml spectinomycin (Spc). The β-lactam concentrations used to select mosaic *pbp2x* and *pbp1a* are specified in the supplemental material.

E. coli DH5α was transformed by using chemically competent cells (82), and transformants were selected in the presence of 100 μg/ml ampicillin or 50 μg/ml spectinomycin.

### Antibiotic susceptibility.

The MICs of the β-lactam antibiotics were determined by the agar dilution method on D-agar plates supplemented with 3% sheep blood under a natural atmosphere. Cells were grown in C+Y medium to an OD_600_ of 0.3, and after 1,000-fold dilution, 30-μl aliquots were spotted onto D-agar plates containing the appropriate antibiotic. In order to detect minor differences in β-lactam susceptibilities, a narrow range of antibiotic concentrations was used. In detail, for strains R6, R6_C_, R6_M_, R6_MC_, and Hu15, concentration steps of 0.01 μg/ml for oxacillin and 0.012 μg/ml for cefotaxime were used, and for the remaining strains, concentration steps of 0.1 μg/ml for oxacillin and cefotaxime were used. The MICs for S. pneumoniae Hu17 were determined with Etest strips (Oxoid GmbH). The MIC values were obtained after incubation at 37°C for 48 h. Mean values from at least three experiments were used.

### Determination of β-galactosidase activity.

Determination of β-galactosidase activity in strains carrying CiaR-controlled promoters in front of a promoterless E. coli *lacZ* gene was performed as described previously (65). The cultures were grown in C+Y or BHI medium, and β-galactosidase activity was measured at three time points: when the OD_600_ was 0.4 and 0.8 and at the beginning of the stationary phase. The volume of the culture harvested for one measurement was adjusted to contain the equivalent of 2 ml cells at an OD_600_ of 0.8. Specific β-galactosidase activities are expressed in nanomoles of nitrophenol released per minute and milligram of protein. Protein concentrations were determined by the method of Bradford (83). Student's *t* test was applied to determine the significance of the results.

### Detection of penicillin binding proteins.

Preparation of samples, PBP labeling with BocillinFL, and separation of proteins by sodium dodecyl sulfate (SDS)-polyacrylamide gel electrophoresis (PAGE) were carried out as described previously (84). BocillinFL-PBP complexes were visualized by fluorography detection with a FluorImager 595 fluorescence scanner (Molecular Dynamics) at 488 nm.

### Preparation of pneumococcal cell wall.

Pneumococcal cell wall preparation and analysis of muropeptides were carried out as described previously (24). Briefly, pneumococci were cultured in 2 liters of C+Y medium to the mid-exponential growth phase (OD_600_, 0.6 to 0.7) and then harvested and resuspended in 40 ml of ice-cold 50 mM Tris-HCl, pH 7.0. The cell suspension was added dropwise into a flask with 150 ml of boiling 5% SDS, and the sample was boiled for an additional 30 min. The samples were centrifuged at 130,000 × *g* at 25°C, and the pellet was washed with deionized water until it was free of SDS. The lysed cells were disrupted with glass beads, and the sample was treated with DNase I (10 μg/ml) and RNase I (50 μg/ml) for 2 h at 37°C with stirring in 100 mM Tris-HCl, pH 7.5, containing 20 mM MgSO_4_. Trypsin (100 μg/ml) and CaCl_2_ (10 mM) were added, and the sample was incubated overnight at 37°C with stirring. SDS was added to yield a final concentration of 1%, and the samples were boiled for 15 min. The resulting purified cell wall was recovered by ultracentrifugation, washed so that it was free of SDS (see above), and lyophilized.

### Preparation of muropeptides and analysis of peptidoglycan composition.

Experiments for preparation of muropeptides and analysis of the peptidoglycan composition were performed as described previously (24). In brief, secondary cell wall polymers were removed by incubation with 48% hydrofluoric acid for 48 h at 4°C. The resulting PG was recovered by centrifugation, washed, and digested with the muramidase Cellosyl for 48 h at 37°C with stirring. The samples were boiled for 10 min at 100°C, and the muropeptides were reduced with sodium borohydride. Reduced muropeptides were separated by HPLC on a 250- by 4.6-mm 3-μm-particle-size Prontosil 120-3-6 C_18_ AQ reversed-phase column (Bischoff, Leonberg, Germany). The eluted muropeptides were detected by their absorbance at 205 nm and assigned by their retention times.

### DNA manipulations and construction of mutants.

All DNA techniques and the construction of strains are described in the supplemental material.

## Supplementary Material

Supplemental material
