# Supplementary material for: New Aspects of the Interplay between Penicillin Binding Proteins, murM, and the Two-Component System CiaRH of Penicillin-Resistant Streptococcus pneumoniae Serotype 19A Isolates from Hungary
Source: Antimicrob Agents Chemother. 2017 Jun 27;61(7):e00414-17. doi: 10.1128/AAC.00414-17 (PMC5487634; doi:10.1128/AAC.00414-17)
Supplement: Supplemental material [file supp_61_7_e00414-17__index.html]

Supplemental material 

# New Aspects of the Interplay between Penicillin Binding Proteins, *murM*, and the Two-Component System CiaRH of Penicillin-Resistant Streptococcus pneumoniae Serotype 19A Isolates from Hungary

## Supplemental material

- Supplemental file 1 -

  Figures S1 and S2, Tables S1 and S2

  PDF, 585K
