# Supplementary material for: New Aspects of the Interplay between Penicillin Binding Proteins, murM, and the Two-Component System CiaRH of Penicillin-Resistant Streptococcus pneumoniae Serotype 19A Isolates from Hungary
Source: Antimicrob Agents Chemother. 2017 Jun 27;61(7):e00414-17. doi: 10.1128/AAC.00414-17 (PMC5487634; doi:10.1128/AAC.00414-17)
Supplement: Supplemental material [file AAC.00414-17_zac007176324s1.pdf]

SUPPLEMENTAL MATERIAL

New aspects of the interplay between penicillin binding proteins, *murM* and the two component system CiaRH of penicillin-resistant *Streptococcus pneumoniae* serotype 19A isolates from Hungary

Inga Schweizer[1], Sebastian Blättner[1,3], Patrick Maurer[1,4], Katharina Peters[2], Daniela Vollmer[2], Waldemar Vollmer[2], Regine Hakenbeck[1] and Dalia Denapaite[1]*

[1]Department of Microbiology, University of Kaiserslautern, Paul-Ehrlich Straße 24, D-67663 Kaiserslautern, Germany
[2]Centre for Bacterial Cell Biology, Institute for Cell and Molecular Biosciences, Newcastle University, Newcastle upon Tyne NE2 4AX, UK
[3]present address:
Lehrstuhl für Mikrobiologie, Biozentrum der Universität Würzburg, Am Hubland, D-97074 Würzburg, Germany
[4]present address:
Hochschule für Technik und Wirtschaft des Saarlandes, Goebenstrasse 40, D-66117 Saarbrücken, Germany

*corresponding author E-mail: denapait@bio.uni-kl.de

Supplementary Figures S1-S2
Table S1

DNA manipulations and construction of mutants
Table S2
References for supplemental material

PBP2x

```
          01112222222233333333333333444444445555555555556666666666666666666666677777777
          36773455567811123344456678001488991133456677791122223333344557777889901122344
          95250104659116801836784914150413180416425724692623681235718271248183630516405
R6        ISAVTLQRMIAQDEIESTMAAVLAIRTQANSIAILNSTVQLDASSQSDTTAESQQPPVDELNSAKAIAVTLNQTADT
Hu15      ...I.........................................................................
Hu17      ....K..QVLGL..LK.PTSSYSVTGIETS...V.HK.IETN..NPV.S.TDTN.AAI.AIE.VRS....F.E....
Hu19A-6   VAT..VLQVL.LND.KGATSSYSVTGIETSTLV.QQ.I..PNVAN..ESPNDTTESAIETIETV..LVIKFDEITNK
```

PBP1a

```
          00000000000000000011111111122222222333333333333333444444444445555555555555555566666666666666666
          00111122223345567700125679923566688001112235588999014455778990113344577777778800022222233333445
          49036913561316816817645116768156715696781631828357543789355573783906001456793506902478902568037
R6        PLILSSVAIVVFSYNQADILSAIAINNQNVASNNEAHTDEAEISILEIHENGNTSIDKATYHNVDLSNANHTSQFALASLNSMYEGSNENIELNS
Hu15      .....................T........................D.................E..............................
Hu17      ............N........T...............S.QSDVATIDMNISADSVMSQTIHNDPEWTGPKYNTGYTMVTIDAI..DDQGTMD..N
Hu19A-6   QIAVCTFTVMILAFSE.EVINTVTVRELGIVNSDQ..S.QSDVATIDMNISADSVMSQTIHNDPEWTGPKYNTGY.MVTVDAI..DDQGTMD..N
850       QIAVCTFTVMILAFSETEVINTVTVRELGIVNSDRSRSEQSDVATIDMNISADN..SQTIHNDPE.TGP...T...MVTIDA.SQNDHGSM.VS-
```

PBP2b

```
          123444444444444455
          323222233344557803
          053678901269026968
R6        IVETQAYGSFTVQAETEN
Hu15      ..................
Hu19A-6   TIGPAFSVPTAIKVGSDD
Hu17      T.GPAFSVPTAIKVGSDD
```

Fig. S1. Deduced amino acid sequences of PBPs of the penicillin resistant strains *S. pneumoniae* Hu17 and Hungary^{19A}-6 compared to the penicillin sensitive *S. pneumoniae* R6. The positions of the amino acids are indicated vertically in the three rows on top. Only positions with altered residues are shown; residues identical to those in strain R6 (bold letters) are indicated by dots. The transpeptidase domain is highlighted in grey. Alignments were prepared using CLUSTALW.

```
          000000000000000000000000000000000000000000000000000000000000000000001111111111111111111111111111111111111111111111111111
          000000111111112222222223333333444444444455555555666666677778888888890000111111122222233333333333444444444444444567777777
          256789001255571112233582345799235677788903444889012344501490245689941679155677901247812345557789000234445666799311245568
          331333122312331231323133133113213312323333123133213313333331233321332333312313333222313231231313123331233123323333332333
Hu19A-6   TGCGAACTCGGGTACCCAGTTCCAAGAGATGCCTGAAGGGCTGTGCGAATGTTGGTTTCACTAGTCGTCCTCTCAGGTATAGAATGGTAGGGTAGGAAGTGCGGAATCGATGTTAGGGAC
Hu17      ........................................................................................................................
Hu15      ........................................................................................................................
Sm SK616  ..............................................C..............................C.....G..T.........G.A...A.................
Sppn 294  ..............................................C...............................................A.........................
R6        CAACTCACTACAGCGAATACCGTGGTGACAATTCAGGAAAAGACAT.TGATGCACCCATGACGTCATCTTCTCAGAACGAGCT.GATGGCAAAG.A.G.AAAAAGGAGAGAACCGAAAGT

          112222222222222222222222222222222222222222222222222222222222222222222222222222333333
          880111122222333333333344444555555555566666666666777777777788888888899999999999113467
          279456836789001455667724455022466778900234456667011223344501144555912334566778052635
          333333333333131313132332312323323132323231331231313231323331213123231133113231213333
Hu19A-6   GACAACTACCAAGCTGCAAGGTGTAAGAAACTAGTCAAAATGACAGGTTCGAAATTAAAAAAAGATGGAAGAATAACAACTGGT
Hu17      ....................................................................................
Hu15      ....................................................................................
Sm SK616  A.....C......T.............G..T..............AAC...........G..................C.....
Sppn 294  A.....C......T.............G..T..............AAC...........G..................C.....
R6        AGAGGTCGTTGCAGGCTGGCCCACGCAGCGTCCTGAGCCCGTGTC.AAAGACGCGAGGGTTTGACCAAGGATCGGAGCAACTC
```

Fig. S2A

```
                000000000000000000000000000000000000000000000000000000000000000000000000000000000000000000000000000111111111111111111111
                000000000111111111122222222223333333333444444444455555555556666666666777777777788888888889999999999000000000011111111112
                123456789012345678901234567890123456789012345678901234567890123456789012345678901234567890123456789012345678901234567890
Hu19A-6         MYRYQLGIPLSEYDGFVKEHPMVNLLQSSAWEKVKSDWNHERLGVYEGENLLAVASILIKSLPLGYKMFYIPRGPILDYRDTELLKFVLQSIKSYARSKRAVFVTFDPSICLSQHLVNQD
Hu17            ........................................................................................................................
Hu15            ........................................................................................................................
Sppn 294        ........................................................................................................................
Sm SK616        ........................................................................................................................
R6              .....I...TL...Q.....ELA.V.......E...N.Q..KF...RE.K...T.....RT..................G.K...N.AI...........V.............S.I..E

                111111111111111111111111111111111111111111111111111111111111111111111111111111122222222222222222222222222222222222222222
                222222222333333333344444444445555555555666666666677777777778888888888999999999900000000001111111111222222222233333333334
                123456789012345678901234567890123456789012345678901234567890123456789012345678901234567890123456789012345678901234567890
Hu19A-6         KREYPENLAIVEILGQLGVKWSGRTIEMDDTIQPRIQAKIYKENFEEDKLSKSTRQAIRTARNKGLEIQYGGLELLDSFSELMKKTEKRKEIHLRNEAYYKKLLDNFKEDSYITLTSLDV
Hu17            ........................................................................................................................
Hu15            ........................................................................................................................
Sppn 294        .................R......................................................................................................
Sm SK616        ......S....D.......E...Q................................................................................................
R6              .T.F......IDS.Q.M..R...K.E..G.........................K.....................................................DKA....AT...

                222222222222222222222222222222222222222222222222222222222223333333333333333333333333333333333333333333333333333333333333
                444444444555555555566666666667777777777888888888899999999990000000000111111111122222222223333333333444444444455555555556
                123456789012345678901234567890123456789012345678901234567890123456789012345678901234567890123456789012345678901234567890
Hu19A-6         SKRLRELEEQLEKNRVVAEKFNDATRSSKVQENIKEKERLKEEIDFLQGYMNMGKSNIPLAATLSLEFGNTSVNLYAGMDDDFKRYNAPILTWYETARYAFERGMVWQNLGGVENSLNGG
Hu17            ........................................................................................................................
Hu15            ........................................................................................................................
Sppn 294        .........................KP.............E............................T..................................................
Sm SK616        .........................KP.............E............................T..................................................
R6              ...SQ......A...ALE.T.TES..T...EAQK......L..LT...E.IDV.QARV...........T....I.............................................

                3333333333333333333333333333333333333334444444
                6666666667777777777888888888899999999990000000
                1234567890123456789012345678901234567890123456
Hu19A-6         LYQFKEKFNPTIEEYLGEFTMPTHPLYPLLRLALDFRKTLRKKHRK
Hu17            ..............................................
Hu15            ..............................................
Sppn 294        ..............................................
Sm SK616        ..............................................
R6              ..H...........................................
```

Fig. S2B

Fig. S2. MurM of *S. pneumoniae* Hu15 and Hu17. Sequences were compared to those of *S. pneumoniae* R6, and *S. mitis* SK616 and *S. mitis* 294. **A:** Nucleotide sequence. The vertical numbers in the first three rows indicate the codon; Sites 1, 2 and 3 refer to the first, second and third position in the respective codon. **B**: amino acid sequence. The first three lines indicate the amino acid position. Only residues that differ from Hu^{19A}-6 are shown; identical residues those in strain R6 are indicated by dots. The aa sequence (244-274 residues) important for the specificity of MurM catalysed reaction (addition of alanine versus serine) are highlighted in grey. *S. pneumoniae* strains Hu15 and R6, and *S. mitis* SK616 (accession no.: AICR01000111.1; Jensen *et al.,* 2015) are penicillin sensitive; the MIC of *S. pseudopneumoniae* 294 (USA) is not known (accession no.: JVMO00000000.1; Roach *et al.,* 2015).

TABLE S1. Muropeptide composition of *S. pneumoniae* strains

Peak No. / Muropeptide[1]		Relative peak area (%) in strains[2]					
		R6	$R6_{C}$	$R6_{2x}$	$R6_{2xC}$	$R6_{2x1a}$	$R6_{2xC1a}$
1	Tri[Glu]	2.7 ± 0.4	3.1 ± 0.3	2.9 ± 0.4	2.9 ± 0.3	3.1 ± 0.1	3.2 ± 0.4
2	Tri[deAc]	7.8 ± 1.1	7.9 ± 1.3	7.9 ± 2.1	8.3 ± 1.3	8.1 ± 0.9	7.4 ± 0.6
3	Tri	19.7 ± 0.1	17.4 ± 0.2	16.8 ± 0.9	16.9 ± 1.2	16.4 ± 0.2	16.3 ± 0.8
4	Tri(A) or Tetra	2.1 ± 0.3	1.4 ± 0.1	1.0 ± 0.1	1.0 ± 0.0	0.7 ± 0.0	0.7 ± 0.0
5	Penta[deAc]	1.1 ± 0.3	1.3 ± 0.3	1.8 ± 0.6	1.8 ± 0.2	1.8 ± 0.2	1.7 ± 0.4
6	Penta[Gly]	0.8 ± 0.3	0.9 ± 0.3	1.1 ± 0.3	1.1 ± 0.1	0.8 ± 0.2	0.8 ± 0.1
7	Penta	3.2 ± 0.6	3.7 ± 0.6	5.1 ± 0.6	5.0 ± 0.1	4.5 ± 0.8	4.5 ± 0.3
8	Tri(SA)[Glu]	0.9 ± 0.1	0.8 ± 0.1	0.8 ± 0.2	0.9 ± 0.1	0.8 ± 0.0	0.9 ± 0.1
9	Tri(S)	1.2 ± 0.4	1.7 ± 0.0	1.6 ± 0.1	1.6 ± 0.0	2.0 ± 0.0	1.9 ± 0.2
10	Tetra(S)[Glu/deAc]	3.0 ± 0.1	3.6 ± 0.0	3.8 ± 0.1	4.0 ± 0.1	5.1 ± 0.6	4.7 ± 0.2
11	Tetra(A)	0.8 ± 0.2	0.7 ± 0.2	0.7 ± 0.2	0.8 ± 0.3	0.6 ± 0.0	0.7 ± 0.2
12	Tri(SA)	8.0 ± 0.5	8.7 ± 0.3	9.9 ± 1.2	9.9 ± 1.0	9.8 ± 0.2	10.1 ± 1.0
13	Tri(AA)[deAc] and Tetra(S)	1.0 ± 0.1	1.1 ± 0.1	1.3 ± 0.1	1.4 ± 0.0	1.5 ± 0.0	1.3 ± 0.2
14	Tri(AA)	2.8 ± 0.1	2.7 ± 0.1	3.2 ± 0.5	3.2 ± 0.4	2.8 ± 0.1	2.9 ± 0.3
15	Tetra(SA)	0.9 ± 0.2	0.8 ± 0.4	0.9 ± 0.5	0.9 ± 0.4	0.8 ± 0.4	0.6 ± 0.1
16	Penta(SA) and Penta(SA)[-G]	0.9 ± 0.2	1.1 ± 0.3	1.5 ± 0.2	1.5 ± 0.2	1.6 ± 0.4	1.7 ± 0.2
17	TetraTri[deAc]‡	2.7 ± 0.6	2.0 ± 0.1	1.9 ± 0.5	1.8 ± 0.3	1.8 ± 0.1	1.8 ± 0.5
18	TetraTri[deAc]‡	3.3 ± 0.5	2.6 ± 0.1	2.6 ± 0.6	2.6 ± 0.3	2.2 ± 0.0	2.2 ± 0.3
19	TetraTri	9.2 ± 0.4	7.4 ± 0.5	6.0 ± 0.2	5.8 ± 0.0	4.9 ± 0.4	5.3 ± 0.0
20	Tetra(SA)Tri[deAc]‡	1.3 ± 0.1	1.6 ± 0.0	1.7 ± 0.0	1.8 ± 0.3	2.4 ± 0.1	2.4 ± 0.3
21	Tetra(SA)Tri[deAc]‡	2.8 ± 0.0	3.4 ± 0.2	3.4 ± 0.3	3.2 ± 0.3	3.5 ± 0.1	3.4 ± 0.2
22	TetraTri(SA)[Glu,deAc]	0.4 ± 0.4	0.5 ± 0.5	0.4 ± 0.4	0.4 ± 0.4	0.6 ± 0.6	0.4 ± 0.4
23	Tetra(SA)Tri	7.8 ± 0.1	8.2 ± 0.2	6.8 ± 0.5	6.3 ± 0.1	6.5 ± 0.4	6.7 ± 0.2
25	Tetra(AA)Tri	3.9 ± 0.9	3.5 ± 0.8	3.1 ± 0.8	3.1 ± 0.8	3.2 ± 1.2	2.8 ± 0.8
26	TetraTri(SA)	2.2 ± 0.0	2.3 ± 0.0	2.3 ± 0.2	2.5 ± 0.7	2.3 ± 0.5	2.8 ± 0.8
27	TetraTri(SA)$_2$[deAc)]‡	1.9 ± 0.1	2.4 ± 0.1	2.6 ± 0.2	2.4 ± 0.3	2.8 ± 0.1	2.9 ± 0.1
28	TetraTri(AA)‡(SA)‡)[deAc]‡	1.6 ± 0.2	1.8 ± 0.1	2.1 ± 0.3	2.0 ± 0.3	2.4 ± 0.1	2.5 ± 0.3
29	TetraTetraTri[deAc]$_2$ [-GM]‡	4.2 ± 0.0	4.5 ± 0.3	4.7 ± 0.3	4.7 ± 0.1	4.6 ± 0.2	4.9 ± 0.1
30	TetraTri(AA)	0.2 ± 0.2	0.2 ± 0.2	0.3 ± 0.3	0.2 ± 0.2	0.2 ± 0.2	0.2 ± 0.2
31	TetraTri(AA)‡(SA)‡	1.8 ± 0.7	2.8 ± 0.1	2.3 ± 0.3	2.3 ± 0.5	2.3 ± 0.7	2.3 ± 0.6

[1] The peak numbers correspond to those previously published (Bui *et al.*, 2012). Modifications: [Glu], presence of glutamine (iGln) instead of glutamic acid (Glu); Gly, presence of Gly at position 5 of the pentapeptide; [deAc], deacetylation of Glc*N*Ac; [-GM], missing Glc*N*Ac-Mur*N*Ac; [-G], missing Glc*N*Ac; ‡, position of the modification(s) not known.

[2] The values are mean ± variations of two independent PG preparations. The Laura software (Lab Logic Systems Ltd) was used for the calculations. The peak areas were estimated as percentage of all known peaks.

DNA manipulations and construction of mutants

All DNA techniques were carried out as described by Sambrook *et al*. (Sambrook *et al.,* 1989). *E. coli* plasmids were isolated using the QIAprep Spin Miniprep kit (Qiagen). The oligonucleotides used in this study are listed in Table S2 and were obtained from Eurofins Genomics. PCR products were amplified using high-fidelity iProof DNA-polymerase (Bio-Rad) according to the manufacturer's instructions. DNA modifying enzymes were purchased from New England Biolabs or Fermentas (Thermo Scientific), and used as described by the manufacturer. Transfer of the genes $pbp2x_{\mathrm{Hu17}}$, $pbp1a_{\mathrm{Hu17}}$, *ciaH*232, and $murM_{\mathrm{Hu17}}$ to *S. pneumoniae* R6 and Hu15 and derivatives were verified by DNA sequencing. The letters in subscript indicate the presence of Hu17 genes in the order of the transformation steps; for example, $\mathrm{R6}_{\mathrm{2x1aMC}}$ was constructed by introducing successively $pbp2x_{\mathrm{Hu17}}$, $pbp1a_{\mathrm{Hu17}}$, $murM_{\mathrm{Hu17}}$ and *ciaH*232.

Strain $\mathrm{R6}_{\mathrm{M}}$ was constructed by a two-step process using the Janus cassette (Sung *et al.,* 2001). First, the Janus cassette was introduced into *murM* of the streptomycin resistant *S. pneumoniae* $\mathrm{R6}^{\mathrm{strR}}$ carrying the *rpsL41* allele (Salles *et al*., 1992). Introduction of the Janus cassette confers a $\mathrm{Kan}^{\mathrm{R}}\mathrm{Str}^{\mathrm{S}}$ phenotype. In a second step, the Janus cassette was replaced by mosaic $murM_{\mathrm{Hu17}}$ gene resulting in strain $\mathrm{R6}_{\mathrm{M}}$. In detail, two fragments of the MurM gene were amplified by PCR using chromosomal R6 DNA and the oligonucleotide pairs PM263/PM238 and PM246/J2. These two fragments were joined by overlapping PCR with the Janus cassette, which had been amplified using oligonucleotides janus_f/janus_r and chromosomal DNA of CCCO*murM::janus* strain (Sauerbier *et al*., 2012). The resulting fragment *murM*::$\mathrm{kan}^{\mathrm{R}}$-$rpsL^{+}$ (2660 bp) was transformed into $\mathrm{R6}^{\mathrm{strR}}$ followed by screening for kanamycin-resistant, streptomycin-sensitive colonies. These transformants contained a non-functional *murM*. One transformant was subjected to a further transformation using the 2522 bp PCR fragment which was amplified using oligonucleotides PM263/J2 and genomic DNA of strain CCCO$murM_{\mathrm{Hu17}}$. Streptomycin-resistant transformants were selected and the presence of $murM_{\mathrm{Hu17}}$ was verified by PCR and sequencing. The strains $\mathrm{R6}_{\mathrm{2xM}}$ and $\mathrm{R6}_{\mathrm{2x1aM}}$ were constructed as described above.

To introduce the allele *ciaH232* into strains $\mathrm{R6}_{\mathrm{M}}$, $\mathrm{R6}_{\mathrm{2x}}$, $\mathrm{R6}_{\mathrm{2xM}}$, $\mathrm{R6}_{\mathrm{2x1a}}$ and $\mathrm{R6}_{\mathrm{2x1aM}}$, the Janus replacement method was used as described by Müller *et al*. (Müller *et al.,* 2011). Briefly, a *ciaH*::kan^{R}-$rpsL^{+}$ fragment was amplified by PCR using primers ciaH_up_ff/ ciaH_down_rr and genomic DNA of strain RKL161 (Müller *et al.,* 2011). The resulting 3057 bp fragment was used to transform streptomycin resistance derivatives harbouring the *rpsL41* allele. The $\mathrm{Kan}^{\mathrm{R}}$ $\mathrm{Str}^{\mathrm{S}}$ transformants were selected and correct integration was verified by PCR. One transformant served as recipient for the *ciaH232* allele, which was amplified by PCR using oligonucleotides ciaH_up_ff/ ciaH_down_rr and genomic DNA of strain RKL243 (Müller *et al.,* 2011). Streptomycin resistant

transformants were selected and the presence of *ciaH*232 verified by sequencing of the *ciaRH* region.

To ensure that only the PBP2x gene of Hu17 and not flanking regions were transferred into *S. pneumoniae* strains R6 and Hu15, PCR fragments covering *pbp2x*$_{\mathrm{Hu17}}$ and the corresponding flanking regions of the recipient strains R6 or Hu15, respectively, were produced by overlapping PCR. PBP2x$_{\mathrm{Hu17}}$ was amplified from Hu17 using the oligonucleotides PM298/PM299. The upstream fragment of *pbp2x*$_{\mathrm{Hu17}}$ was amplified with the oligonucleotides PM83 and PM297, and the corresponding downstream fragment with oligonucleotides PM300 and PM301 using genomic DNA of R6 respectively of Hu15. The three PCR fragments were purified and joined by overlapping PCR using primers PM83 and PM301. The resulting 4678 bp fragments were used to transform R6 or Hu15 and transformants were selected with cefotaxime concentrations at the MIC values of the recipient (0.017 and 0.020 µg/ml, respectively) in order to avoid secondary mutations that might contribute to the resistance phenotype. Under these conditions, no colonies appeared on the control plates. Cell lysates of 20 transformants from each experiment were screened for the presence of low-affinity PBP2x using Bocillin^{TM}FL and fluorography. Nine transformants of R6 strain and thirteen transformants of Hu15 strain showed the presence of low-affinity PBP2x. One transformant each expressing a low affinity PBP2x (R6$_{2x}$ and Hu15$_{2x}$) was chosen for DNA sequencing (Fig. 3).

To generate the strains carrying the mosaic PBP1a$_{\mathrm{Hu17}}$ gene, a PCR fragment covering *pbp1a*$_{\mathrm{Hu17}}$ was amplified using genomic DNA of strain Hu17 as template and the oligonucleotide pair PM205 and PM180. The 4208 bp PCR product was used to transform the strains followed by cefotaxime selection. The cefotaxime concentrations were slightly above the MIC of recipient strain (R6$_{2x}$ 0.4 µg/ml; Hu15$_{2x}$ 0.9 µg/ml). 20 and 12 transformants from strains R6$_{2x}$ and Hu15$_{2x}$, respectively, were screened for presence of low-affinity PBP1a gene after analysis of PBP-profiles as described above. One transformant containing a low affinity PBP1a was chosen for DNA sequencing of *pbp1a* and used for further experiments (R6$_{2x1a}$ and Hu15$_{2x1a}$; Fig. 4).

Table S2. Oligonucleotides used in this work

Name	Sequence (5'-3')
PM263	ACCTTTTGCACGGGATGTCTGGAAATC
PM238	CGGATCCGATCCATTTCCTCTGGAATAGGTTCCTACTCTCTTTCCTCCAGTATTTTATTAC
PM246	CGTCCAAAAGCATAAGGAAAGGGGCCCGTATATGGCACTAACAACACTCACGAAAGAAGAG
J2	GCTCATATAGTCCGAAAAATTTAAGCTTGC
janus_f	CCTATTCCAGAGGAAATGGATCGGATCCG
janus_r	GGGCCCCTTTCCTTATGCTTTTGGACG
PM298	GATATGAAGTGGACAAAAAGAGTAATCCGTTATGCGACC
PM299	CATATTAGTCTCCTAAAGTTAATGTAATTTTTTTAATGTCC
PM83	ATTTGTCTGACAAGTGCAAGCTGGTC
PM297	GGTCGCATAACGGATTACTCTTTTTGTCCACTTCATATC
PM300	GGACATTAAAAAAATTACATTAACTTTAGGAGACTAATATG
PM301	CAAAAACACCCTGAAACATCAGGGTGCCATTC
PM180	CCAACCCCGACTCTTTAAAAGAGTTGGAGTA
PM205	GTCCTCTTGATTTTTCATCTTTTGG
aad9_for	CTAGCTAGCATCGATTTTCGTTCGTGAATACATG
aad9_rev	CCTAGCTAGCACAAAAAAATTGAAAAAAGTGTTTCC

Restriction sites in primer sequences are underlined.

References for Supplemental Material

Bui NK, Eberhardt A, Vollmer D, Kern T, Bougault C, Tomasz A, Simorre JP, Vollmer W. 2012. Isolation and analysis of cell wall components from *Streptococcus pneumoniae*. Anal Biochem: 421:657-666. doi: 10.1016/j.ab.2011.11.026.

Jensen A, Valdórsson O, Frimodt-Møller N, Hollingshead S, Kilian M. 2015. Commensal streptococci serve as a reservoir for β-lactam resistance genes in *Streptococcus pneumoniae*. Antimicrob Agents Chemother 59:3529-3540. doi: 10.1128/AAC.00429-15.

Müller M, Marx P, Hakenbeck R, Brückner R. 2011. Effect of new alleles of the histidine kinase gene *ciaH* on the activity of the response regulator CiaR in *Streptococcus pneumoniae* R6. Microbiology 157:3104-3112. doi: 10.1099/mic.0.053157-0.

Roach DJ, Burton JN, Lee C, Stackhouse B, Butler-Wu SM, Cookson BT, Shendure J, Salipante SJ. 2015. A Year of Infection in the Intensive Care Unit: Prospective Whole Genome Sequencing of Bacterial Clinical Isolates Reveals Cryptic Transmissions and Novel Microbiota. PLoS Genet 11(7): e1005413. doi: 10.1371/journal.pgen.1005413.

Salles C, Créancier L, Claverys JP, Méjean V. 1992. The high level streptomycin resistance gene from *Streptococcus pneumoniae* is a homologue of the ribosomal protein S12 gene from *Escherichia coli*. Nucleic Acids Res 20:6103.

Sambrook J, Fritsch EF, Maniatis T. 1989. Molecular Cloning: a Laboratory Manual, 2nd ed. Cold Spring Harbor, NY: Cold Spring Harbor Laboratory Press.

Sauerbier J, Maurer P, Rieger M, Hakenbeck R. 2012. *Streptococcus pneumoniae* R6 interspecies transformation: genetic analysis of penicillin resistance determinants and genome-wide recombination events. Mol Microbiol 86:692-706. doi: 10.1111/mmi.12009.

Sung CK, Li H, Claverys JP, Morrison DA. 2001. An *rpsL* cassette, Janus, for gene replacement through negative selection in *Streptococcus pneumoniae*. Appl Environ Microbiol 67:5190-5196. doi: 10.1128/AEM.67.11.5190-5196.2001.
